# Dual-energy CT angiography reveals high prevalence of perfusion defects unrelated to pulmonary embolism in COVID-19 lesions

**DOI:** 10.1186/s13244-021-00972-0

**Published:** 2021-02-17

**Authors:** Alice Le Berre, Tom Boeken, Caroline Caramella, Daniel Afonso, Caroline Nhy, Laetitia Saccenti, Anne-Marie Tardivel, Sophie Gerber, Adrien Frison Roche, Joseph Emmerich, Valeria Marini, Marc Zins, Sarah Toledano

**Affiliations:** 1 Department of Radiology, Fondation Hôpital Saint Joseph, 185 rue Raymond Losserand, 75014 Paris, France; 2 Department of Radiology, Hôpital Marie Lannelongue, 133 Avenue de la Résistance, 92350 Le Plessis-Robinson, France; 3 Department of Vascular Medicine, Fondation Hôpital Saint Joseph, 185 rue Raymond Losserand, 75014 Paris, France

**Keywords:** COVID-19 pneumonia, Dual-energy computed tomography, Computed tomography angiography

## Abstract

**Background:**

Lung perfusion defects (PDs) have been described in COVID-19 using dual-energy computed tomography pulmonary angiography (DE-CTPA). We assessed the prevalence and characteristics of PDs in COVID-19 patients with suspected pulmonary embolism (PE) and negative CTPA.

**Methods:**

This retrospective study included COVID-19 and non-COVID-19 pneumonia groups of patients with DE-CTPA negative for PE. Two radiologists rated the presence of PD within the lung opacities and analyzed the type of lung opacities and PD pattern (i.e. homogeneous or heterogeneous). The clinical, biological, radiological characteristics including time from first symptoms and admission to DE-CTPA, oxygen requirements, CRP, D-dimer levels, duration of hospital admission and death were compared within the COVID-19 group between patients with (PD +) or without PD (PD-).

**Results:**

67 COVID-19 and 79 non-COVID-19 patients were included. PDs were more frequent in the COVID-19 than in the non-COVID-19 group (59.7% and 26.6% respectively, *p* < 0.001). Patterns of PDs were different, with COVID-19 patients exhibiting heterogenous PDs (38/40, 95%) whereas non-COVID-19 patients showed mostly homogeneous perfusion defects (7/21 heterogeneous PDs, 33%), *p* < 0.001. In COVID-19 patients, most consolidations (9/10, 90%) exhibited PDs while less than a third of consolidations (19/67, 28%) had PDs in non-COVID-19 patients. D-dimer, oxygen levels and outcome were similar between COVID-19 PD + and PD- patients; however, time between admission and DE-CTPA was longer in PD + patients (median [IQR], 1 [0–7] and 0 [0–2]; *p* = 0.045).

**Conclusion:**

Unlike in bacterial pneumonia, heterogeneous PDs within lung opacities are a frequent feature of COVID-19 pneumonia in PE-suspected patients.

## Key points

1. Perfusion defects unrelated to pulmonary embolism can be detected using dual-energy computed tomography pulmonary angiography; they are more frequent in COVID-19 pneumonia as compared to other lung infections.
2. In COVID-19 patients, perfusion defects exhibit frequently a heterogeneous pattern, whereas bacterial pneumonias usually demonstrate a homogeneous perfusion defect pattern.
3. In COVID-19 patients, perfusion defects are not associated with higher D-dimer levels, oxygen requirements or worse outcome.

## Background

The severe acute respiratory distress syndrome-associated coronavirus-2 (SARS-Cov-2) was first identified in Wuhan, Hubei, China in December 2019 [1]. Soon afterward, progressive respiratory failure and a systemic coagulopathy were found to be critical aspects of the morbidity and mortality in the coronavirus disease 2019 (COVID-19) pandemic [2–4]. The elevation of the D-dimer level is now a widely published biological feature of the disease [5], linked to both inflammatory syndrome and hypercoagulable state [4]. Several publications have also suggested a high prevalence of pulmonary embolism (PE) in COVID-19 patients (2.6%—30%) [6–11]. However, increased D-dimer levels are seen even in the absence of PE.

Interestingly, in the acute respiratory distress syndrome linked to COVID-19, lung compliance seems to be unusually preserved, suggesting that perfusion abnormalities might participate in the occurrence of severe hypoxemia along with the alveolar damage [12]. Corroborating this hypothesis, several autopsy series reported distinctive vascular characteristics associated with COVID-19, including endothelialitis and capillary microthrombi, which were not seen in other conditions such as influenza, along with more anticipated features such as exudative diffuse alveolar damage and surimposed bronchopneumonia [13–15]. Lung perfusion defects (PDs), which could be a radiological correlate of these histological findings, have previously been described in 24 out of 25 patients with COVID-19 using dual-energy computed tomography pulmonary angiography (DE-CTPA) [16, 17]. In light of these recent biological, histological and radiological data, we hypothesized that PDs could be a distinctive radiological finding of COVID-19 related pneumonia. However, these pulmonary vascular manifestations in COVID-19 have not yet been compared to non-COVID-19 lung infections. The relationship between the occurrence of PE-unrelated perfusion abnormalities, perhaps due to distal microthrombosis, and the elevation of the D-dimer level is also unsolved.

Here, we aimed to assess the prevalence and characteristics of perfusion defects unrelated to PE in COVID-19, by comparing patients with COVID-19 and non-COVID-19 community acquired pneumonia with suspected PE and a CTPA negative for PE. Secondary objectives were to investigate the clinical and biological parameters associated with the occurrence PDs unrelated to PE in COVID-19.

## Material and methods

### Patients and study design

This retrospective, monocentric study included a COVID-19 pneumonia group and a non-COVID-19 pneumonia group. This study was approved by our institutional review board. Patients were informed by letter and were offered the possibility not to participate. It followed the ethical guidelines of the declaration of Helsinki.

We identified eligible patients by searching the radiological information system (Xplore, EDL, France) between March and May 2020 for the COVID-19 group and between January 2017 and September 2019 for the non-COVID-19 group.

Patients were included according to the following inclusion criteria:

- in the COVID-19 group:
  - patients over 18 years of age
  - admitted for acute COVID-19 pneumonia and who underwent DE-CTPA at baseline or during hospitalization for suspected PE, defined by suggestive clinical symptoms: respiratory distress, tachycardia, syncope or respiratory worsening
  - diagnosis of SARS-CoV-2 infection confirmed by reverse-transcriptase–polymerase-chain-reaction (RT-PCR) assay
- in the non-COVID-19 group:
  - patients over 18 years of age
  - DE-CTPA done for suspected PE
  - diagnosis of pneumonia on the chest CT report, clinical and biological findings consistent with pulmonary infection (fever, cough, high white blood cell count and C-Reactive Protein (CRP) levels), no evidence for an alternative diagnosis and use of an anti-infective therapy when appropriate (all of the above)

Exclusion criteria for both groups were: CTPA positive for PE, extensive emphysematous changes preventing accurate pulmonary perfusion evaluation.

### Radiological data

#### Image acquisition and post-processing

All DE-CTPA scans were acquired using a 256-slice multidetector CT (Revolution, GE Healthcare, Boston, MA) after intravenous injection of 60 ml iodinated contrast agent (Iomeprol 350 mg I/mL, Bracco, Milan, Italy) at a flow rate of 4 mL/s, using a bolus-tracking technique and a threshold of 100 HU in the main pulmonary artery. The dual-energy acquisition method with the GE scanner involves a rapid kilovoltage-switching technique, in which a single x-ray tube switches the tube energy every 0.5 ms from 80 to 140 kV during the same tube rotation. Other scan parameters were as follows: average tube current 300 mAs, rotation time 0.6–0.8 s, collimation 0.63 mm / 80 mm, pitch 0.992, reconstructed slice-thickness and interval 1.25 mm. Iodine maps, representing water subtraction and allowing quantification of iodine distribution, were reconstructed using the Gemstone Spectral Imaging (GSI) software on an advanced workstation 4.6 (AW 4.6; GE Healthcare).

### Image analysis

All scans were reviewed independently by two radiologists with 3 years’ experience in reading DE-CTPA images in their every-day practice on a PACS workstation (Carestream Health, Rochester, NY). Readers were blinded from the final diagnosis of COVID-19 or non-COVID-19 pneumonia. For each patient, readers rated the presence or absence of perfusion defect within the lung opacities. As in histological studies, we decided to focus on the perfusion changes within the pulmonary lesions rather than on the normal appearing parenchyma. Therefore, the mosaic perfusion pattern, known to be associated with COVID-19 pneumonia [17], was not specifically assessed in this study. Second, readers analyzed the main parenchymal lesion types (ground glass opacity, consolidation, organizing pneumonia’s suggestive features or crazy paving) based on the Fleischner society’s lexicon [18] for each patient. Morphologic features suggestive of organizing pneumonia were perilobular, bandlike subpleural consolidations and the reversed halo sign. Care was taken to exclude areas of atelectasis from our analysis of perfusion defects as they are know to exhibit high iodine content [19]. Each time a PD was detected, the readers qualified it as regionally heterogeneous pattern, when the low iodine distribution areas were patchy and partially matching the lung opacities, or as homogeneous pattern, when the entire pulmonary lesion showed negligible perfusion. In COVID-19 patients, the extent of PDs was rated from 1 to 20 depending on the number of affected pulmonary segments. A 3-point hypoperfusion ratio to COVID-19 lesions was defined as follows: low when PDs involved less than 10% of the lung opacities, moderate between 10 and 30%, and high above 30%.

A perfusion defect was defined as a non-ambiguous area of low iodine distribution within pulmonary lesions (in whole or in part), demonstrating hypoattenuation on the iodine map and hyperattenuation on conventional lung images. PD detection was qualitative. Readers measured the iodine concentration levels on the iodine map (in mg/cm^3^) in order to help patient’s classification; although no arbitrary threshold was chosen because of the lack of previous evidence and vendor recommendation in that matter. Additionally, a quantitative assessment of the iodine concentration of the normal appearing parenchyma was measured in mg/cm^3^ by placing a 1 cm^2^ region-of-interest in a disease-free area, with care taken to avoid pulmonary vessels, in order to assess the comparability between the scans with and without PD.

In COVID-19 patients, the extent of the disease was also visually assessed using a 5-point scale: < 10% (limited), 10–25% (mild), 25-–0% (moderate), 50–75% (severe), > 75% (diffuse) [20].

Any discrepancies between the two radiologists were resolved through discussion, until consensus was reached.

### Clinical and laboratory data

All data were extracted from our computerized medical records (DX Care, Dedalus, France). Demographic characteristics including age and sex were recorded in both groups. For COVID-19 patients, the following data were recorded: time from onset of symptoms (fever, cough, shortness of breath, tiredness, diarrhea) and hospital admission to DE-CTPA; antithrombotic treatment before DE-CTPA; maximal and at DE-CTPA oxygen requirements; need for invasive mechanical ventilation; maximal and at DE-CTPA D-dimer levels; highest value of C-reactive protein (CRP); and duration of hospital admission (for alive patients). For non-COVID-19 patients, the established or suspected type of infectious pneumonia (bacterial, viral, fungal for the main types) was obtained from medical records, taking into account the CT findings, the type of anti-infectious therapy instituted and its outcome when the infection was not microbiologically documented. We specified the infectious agent involved when identified (through urinary antigen test, PCR, blood or sputum culture).

### Study outcomes

The main purpose of our study was to compare the prevalence of PDs unrelated to PE in COVID-19 as compared to a group of non-COVID-19 community-acquired pneumonia. Secondary objectives were to identify clinical, radiological or biological characteristics and outcome associated with the presence of PDs in COVID-19 patients.

### Statistical analysis

Normality was tested using the Shapiro–Wilk test. Normally distributed values, expressed as mean (± standard deviation), were compared with two-tailed Student’s t-tests; non-normally distributed values, expressed as median (interquartile range (IQR)), were compared with Mann–Whitney U-tests. Categorial parameters were compared with χ2 or Fisher’s tests when appropriate. Relationships between hypoperfusion extent and ratio to D-dimer and O_2_ levels were assessed using Pearson correlation. A *p* value < 0.05 was considered significant. All statistical analyses were performed using the XLSTAT v2018.7 software.

## Results

### Patients

**Fig. 1:**
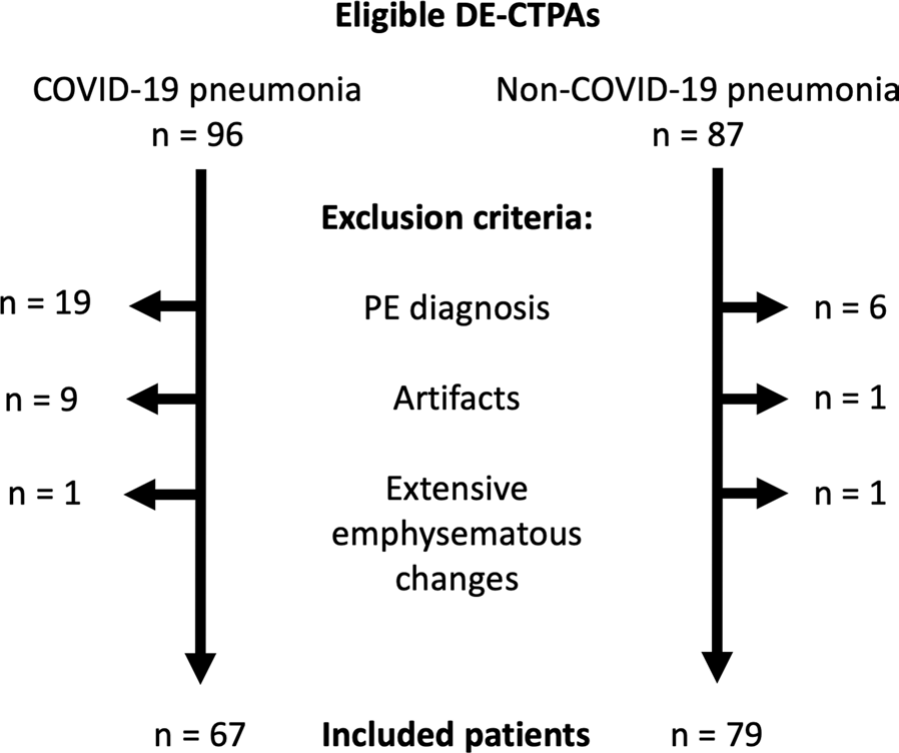
Study’s flow chart. DE-CTPA: dual-energy computed tomography pulmonary angiography, PE: pulmonary embolism

Ninety-six and 87 respectively COVID-19 and non COVID-19 potentially eligible patients were identified by our database search. After application of the exclusion criteria, 29 and 8 respectively COVID-19 and non-COVID-19 cases were excluded from the study, for reasons given in Fig. 1. The remaining 67 COVID-19 and 79 non-COVID-19 patients were included. There were 43 males and 24 females (age 64 [55–76], median [IQR]) in the COVID-19 group and 34 males and 45 females (age 71 [55–82]) in the non-COVID-19 group. Table 1 summarizes the different types of pneumonia included in the non-COVID-19 group. Most of them were suspected or documented bacterial pneumonias. The infection agent was only identified in 20 cases.

**Table 1:** Pneumonia types included in the non-COVID group

Pneumonia type	Diagnosis of infection	Subtype or infectious agent	*n*	PD + /PD-
Bacterial	Suspected	Aspiration pneumonia	6	1/5
		Superinfection post-influenza	2	1/1
		Other	47	14/33
	Documented	*Pneumococcus*	7	3/4
		*Legionella pneumophilia*	4	0/3
		*Pseudomonas aeruginosa*	4	1/3
		*Klebsiella pneumoniae*	1	0/1
		*Staphylococcus aureus*	1	0/1
		*Mycobacterium tuberculosis*	1	1/1
Viral	Suspected		1	0/1
Fungal	Documented	*Aspergillus fumigatus*	1	0/1
		*Pneumocystis jiroveci*	1	0/1
Indeterminate			3	0/3

When microbiologically documented, the infectious agent implicated was noted

PD + : patients with perfusion defects; PD-: patients without perfusion defects

### Prevalence and characteristics of perfusion defects

Perfusion defects were more frequent in the COVID-19 group than in the non-COVID-19 group (n = 40/67, 59.7% and n = 21/79, 26.6% respectively, *p* < 0.001). Considering all subjects, the mean iodine attenuations in the disease-free areas were similar between patients with (PD +) or without (PD-) PDs (0.785 and 0.698 mg/cm^3^, *p* = 0.09) asserting comparability between scans. Mean iodine attenuation measured in PDs was 0.358 ± 0.284 mg/cm^3^ (mean ± standard derivation).

Patterns of PDs were significantly different, with COVID-19 patients exhibiting mostly heterogenous PDs (38/40, 95%) whereas non-COVID-19 patients showed mostly homogeneous perfusion defects (7/21 heterogeneous PDs, 33%), *p* < 0.001.

Consolidations were depicted in 10/67 cases in the COVID-19 group and in 67/79 cases in the non-COVID-19 group. However, in the COVID-19 group, most consolidations (9/10, 90%) exhibited PDs while less than a third of consolidations (19/67, 28%) had PDs in the non-COVID-19 group. Ground glass opacities demonstrated low iodine concentration in respectively 20 and 25% in the COVID-19 and non-COVID-19 groups. (Table 2).

**Table 2:** Perfusion defects seen in COVID-19 and non-COVID-19 pneumonias depending on the type of parenchymal lesion

	Pneumonia type			Lesion types		
		GGO	CS	OP	CP	TIB
Perfusion defects (n/total, %)	COVID-19	3/15 (20%)	9/10 (90%)	18/22 (82%)	10/20 (50%)	0/0 (0%)
	Non-COVID-19	2/8 (25%)	19/67 (28%)	0/1 (0%)	0/1 (0%)	0/2 (0%)

The main lesion exhibiting a perfusion defect in PD + patients or the most represented lesion type in PD- patients were recorded

GGO: ground glass opacity; CS: consolidation; OP: organizing pneumonia’s suggestive features; CP: crazy-paving; TIB: tree-in-bud; PD + : presence of a perfusion defect; PD-: absence of perfusion defect

Crazy paving and morphologic features of organizing pneumonia were seen in respectively 20 and 22 cases in the COVID-19 group while it was rare in the non-COVID-19 group (1 case each). A total of 10/20 crazy paving (50%) and 18/22 (82%) organizing pneumonia exhibited perfusion defects in the COVID-19 group.

**Fig. 2:**
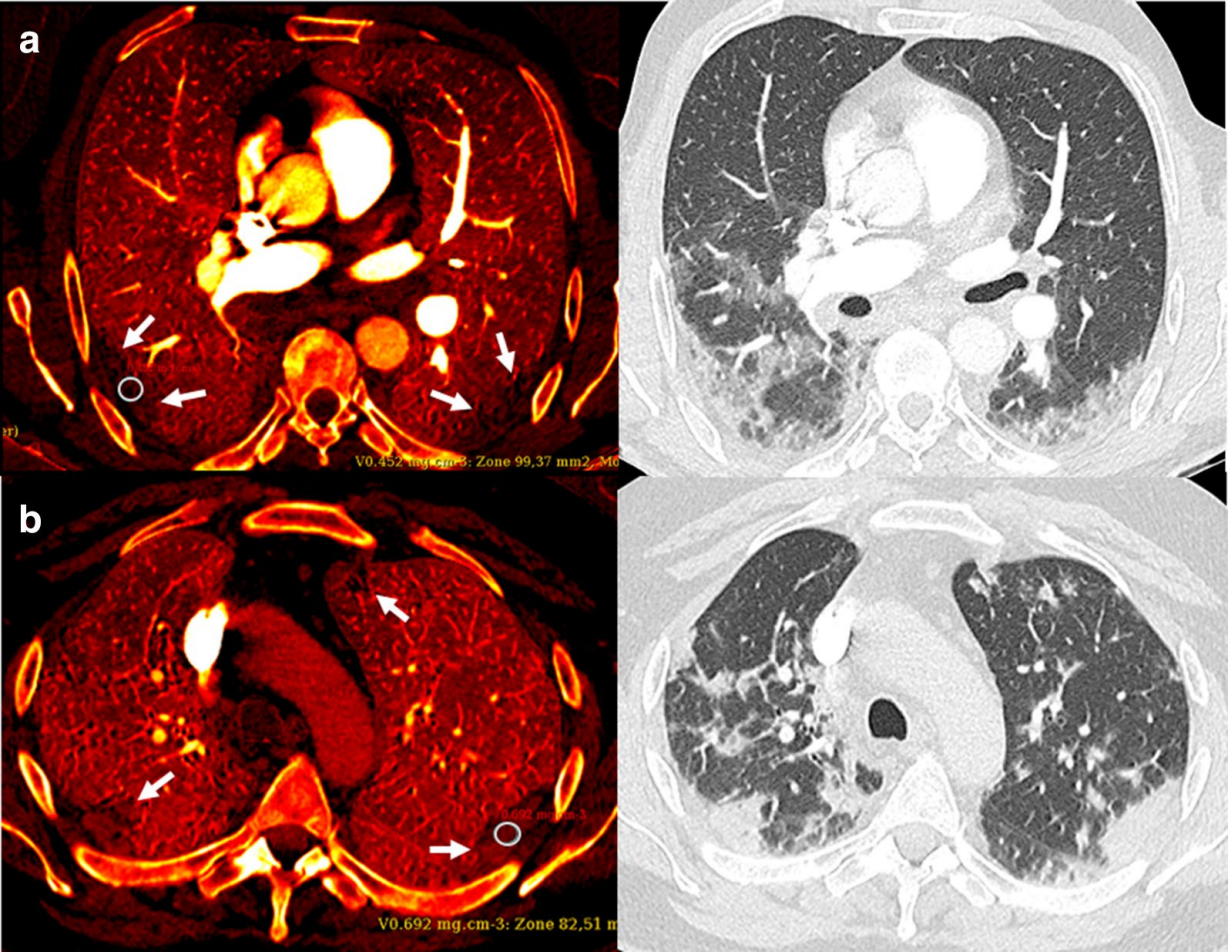
**a** 66-year-old male with COVID-19 related pneumonia at hospital day 7. A rt-PCR assay confirmed the diagnosis. DE-CTPA was performed for increased D-dimer (640 ng/mL) and oxygen requirement levels. Heterogeneous iodine distribution (PD +) is seen with sub-pleural perfusion defects (iodine map, left) partially matching the consolidations in the lower lobes (conventional lung images, right). Iodine concentration was measured at 0.452 mg/cm^3^ using a 1 cm^2^ ROI within the circle and at 0.873 mg/cm^3^ within the normal-appearing parenchyma. **b** 57-year-old-male with a rt-PCR confirmed COVID-19 pneumonia also at hospital day 7. The D-dimer level was increased at 1160 ng/mL. Heterogeneous perfusion defects (PD +) are seen in the sub-pleural areas (iodine map, left), partially matching the lung opacities (right). Iodine concentration was measured at 0.692 mg/cm^3^ within the circle and at 0.928 mg/cm^3^ within the normal-appearing parenchyma

**Fig. 3:**
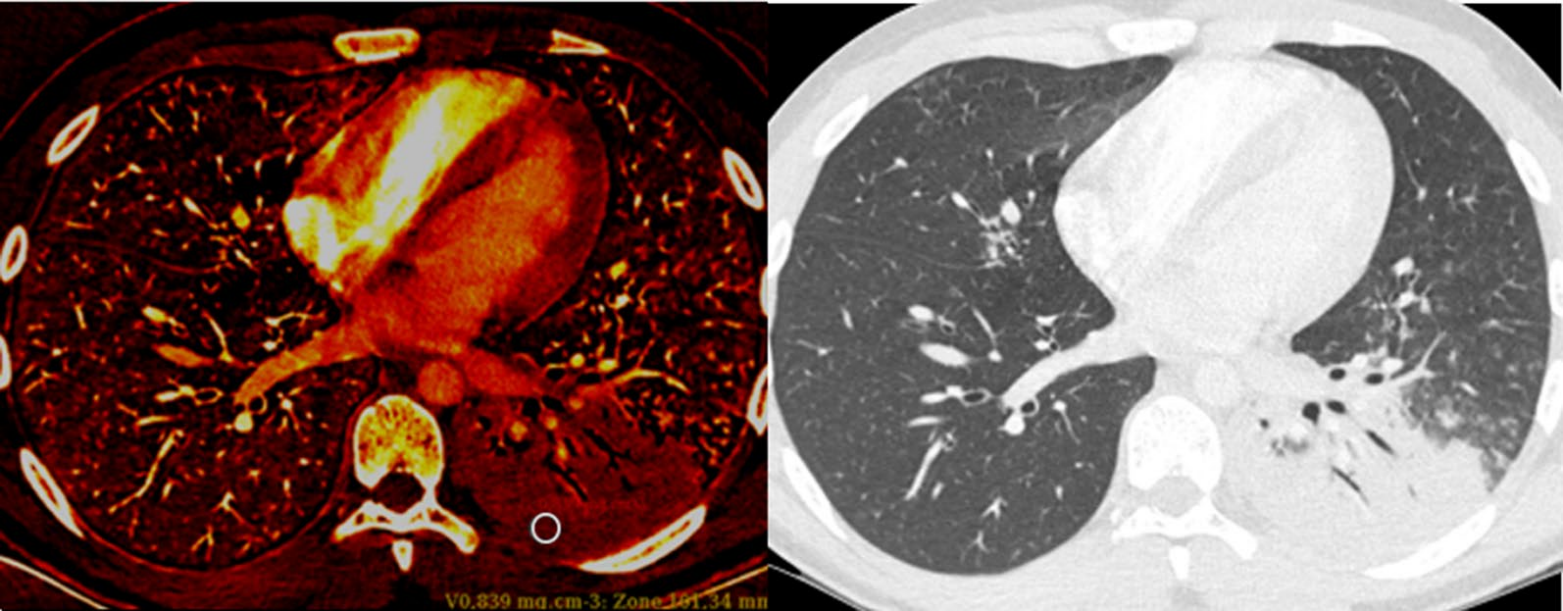
19-year-old male with hemoptysis and chest pain. A homogeneous perfusion defect (PD +) is seen (iodine map, left) matching a left basal consolidation (conventional lung images, right). Tuberculous pneumonia was confirmed by PCR assay performed a on sputum sample. Iodine concentration was measured at 0.839 mg/cm^3^ within the circle and at 0.686 mg/cm^3^ within the normal-appearing parenchyma

**Fig. 4:**
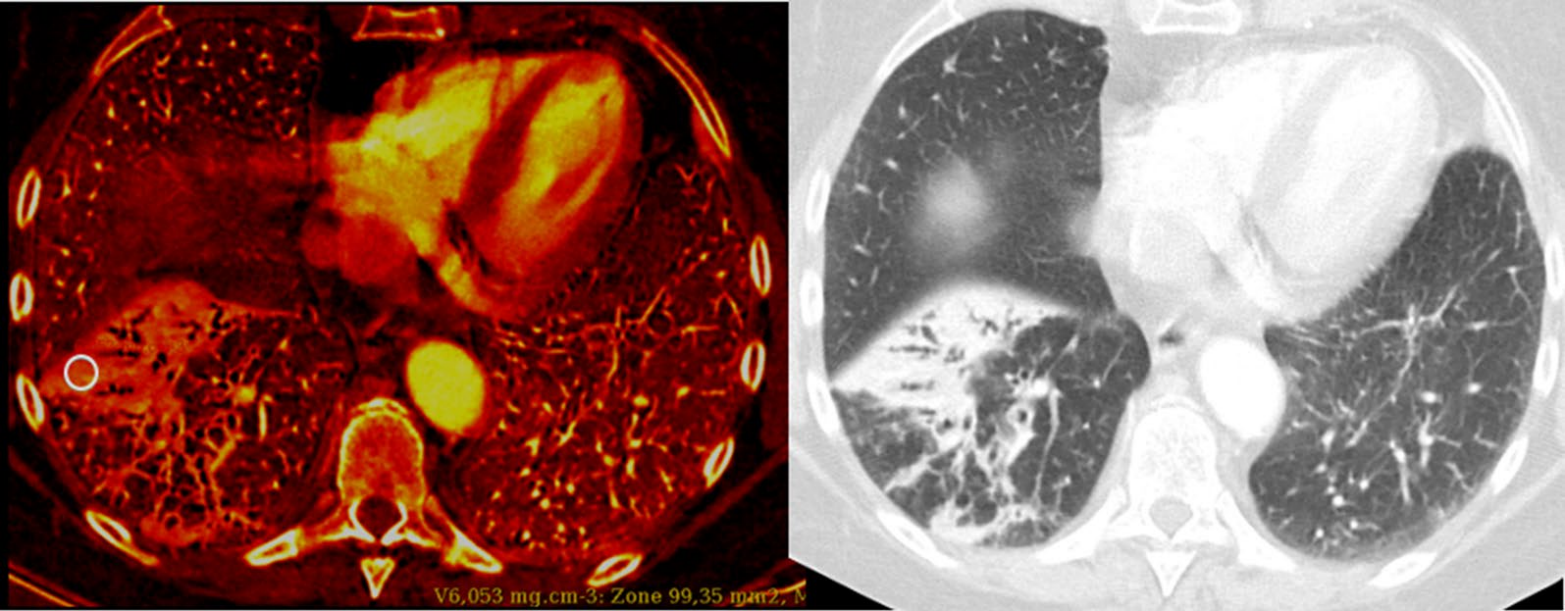
75-year-old female with bronchopneumonia and suspected PE. No perfusion defect was detected (PD-). Instead, a homogeneous high iodine distribution is seen (iodine map, left) matching consolidations in the right anterior basal segment (conventional lung images, right). The patient was treated with Ceftriaxone with a favorable outcome. Iodine concentration was measured at 6.053 mg/cm^3^ within the circle and at 0.723 mg/cm^3^ within the normal-appearing parenchyma

Three selected cases are illustrated in Figs. 2, 3 and 4.

Characteristics and outcomes of COVID-19 patients in PD + and PD- groups (Table 3).

**Table 3:** Clinical, biological, radiological characteristics and outcome of PD + and PD- COVID-19 patients

Characteristics	Perfusion defect + (n = 40)	Perfusion defect -(n = 27)	*p* value
Age (median, IQR)—yr	64 (55–76)	66 (56–84)	0.494
Male sex—no. (%)	24 (60)	19 (48)	0.444
Time from onset of symptoms to DE-CTPA, days (median, IQR)	14 (7–18)	10 (7–14.5)	0.130
Time from admission to DE-CTPA, days (median, IQR)	1 (0–7)	0 (0–2)	0.045
Disease extent assessed on CT lung images			
< 10%—no. (%) *(limited extent)*	1 (3)	3 (11)	0.295
10–50%—no. (%) *(regrouping mild and moderate extent)*	17 (43)	13 (48)	0.799
> 50%—no. (%) *(regrouping severe and diffuse extent)*	22 (55)	11 (41)	0.321
Anticoagulant treatment prior to DE-CTPA			
Preventive dosage—no. (%)	13 (33)	8 (30)	1
Curative dosage—no. (%)	5 (13)	2 (7)	0.693
Oxygen-support at DE-CTPA			
Invasive ventilation- no. (%)	0 (0)	1 (4)	0.023
High-flow oxygen (≥ 6 l/min)—no. (%)	14 (35)	7 (26)	0.592
Low-flow oxygen (< 6 l/min and room air)—no. (%)	26 (65)	19 (70)	0.792
Highest oxygen-support during hospital stay			
Invasive ventilation- no. (%)	9 (23)	3 (11)	0.335
High-flow oxygen (≥ 6 l/min)—no. (%)	14 (35)	9 (33)	1
Low-flow oxygen (< 6 l/min and room air)—no. (%)	17 (43)	15 (56)	0.328
Biological characteristics			
D-Dimer level just prior to CTPA—median (IQR) ng/mL	1340 (910–2625)	1590 (1140–2142)	0.340
Highest level of D-dimer during hospitalization – median (IQR) mg/L	1760 (1173–4085)	2221 (1445–3433)	0.305
Highest level of CRP during hospitalization – median (IQR) mg/L	146 (75–194)	135 (62–206)	0.944
Prognosis			
Duration of hospital admission (alive patients)- days (median, IQR)	14 (8–23)	9 (4–17)	0.117
Deaths—no. (%)	3 (8)	6 (22)	0.142

The median time between onset of symptoms and DE-CTPA were similar between the two groups, but the median time between admission and DE-CTPA was longer in the PD + group (median [IQR], 1 [0–7] and 0 [0–2]; *p* = 0.045). Anticoagulant treatments prior to DE-CTPA were comparable and there was no difference in terms of CRP, D-dimer or oxygen levels between PD + and PD- groups. Disease extent on CT lung images was similar.

No difference regarding outcome was noted: patients with and without PD had similar length of total hospital stay, and death.

### *Perfusion defects extension in PD* + *COVID-19 patients*

In COVID-19 patients exhibiting PDs, a median of 4 (inter-quartile range 2–5) pulmonary segments were affected. The hypoperfusion ratio to COVID-19 lesions was low (< 10%), moderate (10–30%) and high (> 30%) in 12, 12 and 16 patients, respectively. Hypoperfusion extent and ratio were not significantly correlated to neither D-dimer nor oxygen levels.

## Discussion

Our retrospective study comparing DE-CTPA scans negative for PE of COVID-19 and non-COVID-19 pneumonias found a higher prevalence of perfusion defects within lung opacities in COVID-19 patients. This finding is consistent with the first published series of DE-CTPA in COVID-19 [17] reporting a very high prevalence of oligemia; however, our study included more COVID-19 patients with DE-CTPA scans (67 versus 25) and a control group. These differences in terms of lung perfusion do not seem solely explained by the different types of parenchymal lesions observed between COVID-19 and non-COVID-19 pneumonia. Indeed, consolidations exhibited perfusion defects in the COVID-19 group more frequently than in the non-COVID-19 group (90% versus 28%), suggesting an underlying pathological process specific to COVID-19.

To avoid confusion with perfusion defects linked to macro-thrombosis and embolism, we excluded PE patients from the study. As might have been expected considering the high prevalence of PE in COVID-19 [6–9], a greater number of PE patients were excluded from the COVID-19 group than from the non-COVID-19 group. The male predominance, known to be a feature of the disease is also reflected in our study sample (64%) [21]. The PD pattern was more frequently heterogenous in the COVID-19 group than in the non-COVID-19 group. This could perhaps be due to the wider spectrum of lesion types within each patient individually in COVID-19, whereas lesion types were more uniform in community acquired pneumonia. Unlike Lang et al. [17], we did not observe any “hyperemic halo” surrounding oligemic pulmonary opacities (found in 9 out of 25 patients). The use of different scanners between our two studies might have played a role in these differences as this finding was illustrated on pulmonary blood volumes (PBV) images obtained using a scanner from a different vendor.

By acquiring near-simultaneous CT images at two x-ray energy levels [22], DE-CTPA allows the decomposition of images into two base materials (e.g., iodine and water) and thereby separate regions of iodine uptake from unenhanced soft tissues. Hence, iodine maps can be obtained to demonstrate the distribution of pulmonary perfusion [23]. The main clinical application of DE-CTPA is the evaluation of pulmonary embolism (PE) [24]. The spectrum of pulmonary infections has been far less explored. Some authors described increased or decreased heterogenous iodine distribution in pneumonia [19, 25], matching the abnormalities seen on conventional lung attenuation images, as opposed to pulmonary embolism in which the mismatch between perfusion-ventilation images is a key diagnostic feature. However, to our knowledge, the physiopathological significance and clinical implication of these findings have not been further investigated.

We observed a homogeneous hypoperfusion pattern in a majority of PD + non-COVID patients. Little is known about the perfusion profile of community acquired pneumonias on DE-CTPA and it was out of the scope of this study. However; we hypothesize that hypoperfusion, less frequent than in COVID-19, could be associated with suppurative or necrotizing forms (i.e. in pneumococcal or tuberculous pneumonia), but this topic would require further specific research. Another important finding of the study is that PDs were not associated with a worse outcome in terms of oxygen requirement, need for invasive ventilation, length of hospitalization nor death. The D-dimer level and anticoagulant therapy prior DE-CTPA were also similar between PD + and PD- patients. The extent of PDs was not correlated to the D-dimer level nor to the patients’ oxygen requirements. According to these findings, PDs could therefore be seen as a feature suggestive of COVID-19 pneumonia, along with other typical CT findings [26], rather than a marker of severity.

Several recent autopsy studies have described widespread vascular thrombosis with microangiopathy and occlusion of alveolar capillaries in post-mortem COVID-19 lungs [13, 14]. Menter et al. reported alveolar capillary microthrombi 9 times as prevalent in patients with Covid-19 as in patients with influenza. Furthermore, clinically, many patients have elevated D-dimer levels [5], as well as cutaneous changes in their extremities, suggesting thrombotic microangiopathy [27] and generalized thrombotic microvascular injury. These distinctive pathology vascular findings substantiate the perfusion patterns we observed on DE-CTPA and support the global hypothesis of the impact of diffuse vascular abnormalities in COVID-19 pneumonia. However, the exact nature of these PDs (e.g. micro-emboli, in-situ thrombosis, or vasocontriction) remains unelucidated.

This study has several limitations. First, the inclusion of patients who underwent DE-CTPA for suspect PE automatically induces a selection bias. In the COVID-19 group, DE-CTPAs were performed when the clinical state of patients worsened, the study sample therefore included more severe patients and did not reflect the entire spectrum of COVID-19 pneumonia. Also, during the first epidemic wave, the selection criteria for CTPA may have been stricter in order to minimize staff exposure. The level of D-dimer was also susceptible to be higher in these PE-suspected patients. This bias may have flattened the differences between the PD + and PD- COVID-19 patients. In the non-COVID-19 group, the selection of patients referred for suspected PE resulted in the inclusion of a large majority of patients with bacterial rather than viral pneumonia (only one patient), more susceptible to present with chest pain or D-dimer elevation linked to the inflammatory syndrome. Comparing COVID-19 to other viral infections, exhibiting rather diffuse alveolar damage than suppurative bronchopneumonia, would have been of great interest, but such cases are less prevalent, and patients rarely undergo DE-CTPA, so the data were not available to us. Although it is a major limitation of this study, it wouldn’t have been ethical to collect prospectively DE-CTPA in patients with viral pneumonia and no PE suspicion. Secondly, although the readers were blinded to the diagnosis, COVID-19 CT findings are quite typical and the evaluation of perfusion abnormalities subjective, potentially leading to a misclassification bias. We tried to reduce this bias by establishing a consensus between readers in difficult cases. The use of an iodine concentration threshold would have allowed for a quantitative analysis, but we preferred a subjective evaluation using experts in the absence of a standard criterion and vendor recommendation. Another limitation is the lack of temporal perspective on these DE-CTPA findings: are perfusion defects an early or a tardive feature of the COVID-19 pneumonia? We might think that perfusion defects appear at a later stage since more patients with PD were diagnosed on a follow-up rather than on a baseline DE-CTPA, and occurred in consolidations and organizing pneumonia’s suggestive lesions rather than in ground glass opacities seen at an earlier stage [28], but this would need further investigation. Finally, for clarity purposes, we did not assess the occurrence of hyperemia or hyper-perfusion in this study. This could be a matter of interest in both COVID-19 and non-COVID-19 pneumonias.

Beyond offering novel insights relevant to our understanding of the disease, the clinical application of these findings remains to be determined. DE-CTPA could potentially help diagnosing litigious COVID-19-suspected cases. However, in our opinion the use of DE-CTPA has to be motivated by a clinical PE suspicion.

In conclusion, unlike in bacterial pneumonia, heterogeneous perfusion defects within lung opacities could be a specific feature of COVID-19 pneumonia, in line with previously published histological data.

## Data Availability

The datasets used and/or analysed during the current study are available from the corresponding author on reasonable request.

## Abbreviations

DE-CTPA: Dual-energy computed tomography pulmonary angiography
PD: Perfusion defect
PE: Pulmonary embolism
